# (-)-Oleocanthal Nutraceuticals for Alzheimer’s Disease Amyloid Pathology: Novel Oral Formulations, Therapeutic, and Molecular Insights in 5xFAD Transgenic Mice Model

**DOI:** 10.3390/nu13051702

**Published:** 2021-05-18

**Authors:** Afsana Tajmim, Areli K. Cuevas-Ocampo, Abu Bakar Siddique, Mohammed H. Qusa, Judy Ann King, Khaldoun S. Abdelwahed, Jafrin Jobayer Sonju, Khalid A. El Sayed

**Affiliations:** 1 School of Basic Pharmaceutical and Toxicological Sciences, College of Pharmacy, University of Louisiana at Monroe, 1800 Bienville Drive, Monroe, LA 71201, USA; tajmima@warhawks.ulm.edu (A.T.); siddique@ulm.edu (A.B.S.); qusamh@warhawks.ulm.edu (M.H.Q.); abdelwks@warhawks.ulm.edu (K.S.A.); sonjujj@warhawks.ulm.edu (J.J.S.); 2 Department of Pathology and Translational Pathobiology, LSU Health Shreveport, 1501 Kings Highway, Shreveport, LA 71103, USA; acueva@lsuhsc.edu (A.K.C.-O.); jkin12@lsuhsc.edu (J.A.K.)

**Keywords:** β-amyloid, C3AR1, EVOO, oleocanthal, oral formulation, STAT3, Alzheimer’s disease

## Abstract

Alzheimer’s disease (AD) is a complex progressive neurodegenerative disorder affecting humans mainly through the deposition of Aβ-amyloid (Aβ) fibrils and accumulation of neurofibrillary tangles in the brain. Currently available AD treatments only exhibit symptomatic relief but do not generally intervene with the amyloid and tau pathologies. The extra-virgin olive oil (EVOO) monophenolic secoiridoid *S*-(–)-oleocanthal (OC) showed anti-inflammatory activity through COX system inhibition with potency comparable to the standard non-steroidal anti-inflammatory drug (NSAID) like ibuprofen. OC also showed positive in vitro, in vivo, and clinical therapeutic effects against cardiovascular diseases, many malignancies, and AD. Due to its pungent, astringent, and irritant taste, OC should be formulated in acceptable dosage form before its oral use as a potential nutraceutical. The objective of this study is to develop new OC oral formulations, assess whether they maintained OC activity on the attenuation of β-amyloid pathology in a 5xFAD mouse model upon 4-month oral dosing use. Exploration of potential OC formulations underlying molecular mechanism is also within this study scope. OC powder formulation (OC-PF) and OC-solid dispersion formulation with erythritol (OC-SD) were prepared and characterized using FT-IR spectroscopy, powder X-ray diffraction, and scanning electron microscopy (ScEM) analyses. Both formulations showed an improved OC dissolution profile. OC-PF and OC-SD improved memory deficits of 5xFAD mice in behavioral studies. OC-PF and OC-SD exhibited significant attenuation of the accumulation of Aβ plaques and tau phosphorylation in the brain of 5xFAD female mice. Both formulations markedly suppressed C3AR1 (complement component 3a receptor 1) activity by targeting the downstream marker STAT3. Collectively, these results demonstrate the potential for the application of OC-PF as a prospective nutraceutical or dietary supplement to control the progression of amyloid pathogenesis associated with AD.

## 1. Introduction

Alzheimer’s disease (AD) is manifested as a progressive, multifactorial neurodegenerative brain disorder attributed by the deposition of β-amyloid (Aβ) plaques and the accumulation of neurofibrillary tangles (NFTs) in brains of diseased individuals [1,2]. Approximately 5.8 million Americans aged 65 and older have been affected with Alzheimer’s disease in 2020 [1,3]. Because of rapid growth of the population and ageing, the number of American patients living with AD is expected to reach 14 million by 2050 [4]. AD is considered the sixth leading cause of death and the most common cause of dementia worldwide [3,5]. Unfortunately, despite decades of scientific studies on risk factors, genetic mechanism, detection biomarkers, and therapeutics, there is no satisfying therapy or FDA-approved drug to date, which will terminate or prevent the progression of AD by acting on its pathogenesis origin [5]. Emerging evidence supports that currently available therapeutics only exhibit symptomatic relief but fail to attain a definite cure nor disease prevention [4]. Pathogenesis of AD involves a couple of defined pathways, amyloid-β (Aβ) and tau-related neuropathology [3,4,5]. Amyloid-related brain pathogenesis caused by accumulation and precipitation of Aβ peptides (mainly Aβ_40_ and Aβ_42_) produced by proteolytic degradation of amyloid-β precursor protein (APP) [3,4,5]. Increased Aβ_40_ and Aβ_42_ brain levels promote insoluble plaques formation, which is an established AD pathogenesis signature in patient brains. The second major AD pathway is the tau-associated changes promoted by hyperphosphorylated tau somatodendritic accumulation, blocking the normal tau assembly to microtubules and precipitating neurofibrillary tangles.

The complement system is an important part of the innate immune system, which enhances the antibodies and phagocytic cells to clear damaged cells and pathogenic microbes and promote inflammation [6]. It was also proven to be a disease risk component and response in AD. Typical complement activation occurs by the cleavage of the central complement factor C3 to C3a and C3b, and subsequently bind to their corresponding receptors C3AR and CR3, respectively [7]. Several studies have documented the effect of the C3-C3aR signaling pathway in β-amyloid and tau pathogenesis [8,9,10,11,12]. C3 activates the receptor C3AR1, which expressed as a seven-transmembrane spanning G-protein coupled receptor distributed in brain neurons, astrocytes, and microglia [13]. The C3AR1 is a regulatory hub in the innate immunity system located at the terminal part of the complement system, which regulates chemotaxis and signaling. C3AR1 is upregulated in AD and its selective targeting expected to modulate inflammation without adversely affecting other critical complement processes like phagocytosis and clearance (C3b) and the membrane attack complex (C5b/MAC). Recently, Litvinchuk et al. demonstrated the valuable role of the complement C3AR in the immune network and neuronal tau pathology where tau was decreased through the inactivation of C3AR [14]. The activated STAT3 was defined as a downstream effector of C3AR1-mediated AD pathology [14].

(–)-Oleocanthal (OC, decarboxymethyl ligstroside aglycone) is a naturally occurring phenolic secoiridoid in extra-virgin olive oil (EVOO) with documented therapeutic effects against inflammation, many malignancies, and neurodegenerative diseases, especially AD. OC provides the irritative, bitter, and pharyngeal taste of EVOO and is proven to possess anti-inflammatory effects equivalent to the non-steroidal anti-inflammatory drug ibuprofen [15,16,17,18,19,20,21,22,23]. Numerous studies highlighted the effects of OC against the β-amyloid plaque accumulation and neurofibrillary tangles of hyperphosphorylated tau. These studies clearly validated OC as a promising lead against AD pathology [24,25,26,27,28]. In 2009, Pitt et al. (2009) hypothesized the anti-AD effects of OC through the disruption of Aβ oligomerization, and alteration of the oligomerization state of ADDLs (Aβ-derived diffusible ligands), along with the attenuation of synaptic binding and synaptotoxicity [24]. Similarly, Li et al. investigated the exceptional in vitro neuroprotective effects of OC via inhibition of tau proteins’ fibrillization, whereas this inhibition was not observed with other NSAIDs like ibuprofen [25]. Studies also confirmed the OC capability to inhibit tau protein fibrillization by covalent cross-linking with the ε-amino group of lysine residues in tau fragment K18 [26]. To develop OC as AD therapeutics, Abuznait et al. (2013) and Qosa et al. (2015) further investigated the potential of OC against AD [27,28]. OC induced amyloid β (Aβ) efflux and clearance by up-regulation of the major Aβ transport proteins across the blood–brain barrier (BBB), namely, P-glycoprotein and low-density lipoprotein receptor (LDLR)-related protein-1 (LRP1) in TgSwD1 mice [27,28]. OC has been documented to reduce the astrocyte activation and IL-1β levels in the brain of an AD animal model [28]. OC-enriched EVOO treatments enhanced the expression of IDE and NEP, the Aβ degrading enzymes, and synergized with therapeutic effects of donepezil in the 5xFAD transgenic model [29]. Treatments with OC-enriched EVOO alone or combined with donepezil significantly induced ABCA1, activating PPARγ expression, which can promote P-gp and LRP1 levels, improving the Aβ clearance [29]. OC-enriched EVOO also attenuated extravasation of IgG, and improved memory function in 5xFAD mice. OC restored the in vitro levels of GLT1 and GLUT1 in astrocytes [30]. OC modulated neuro-inflammation and prevented the Aβ-induced synaptic proteins down-regulation (SNAP-25 and PSD-95) [30].

Despite bearing a good potential for cognitive impairment, an ideal pharmaceutical oral dosage form of OC is not available until now for plausible use as a nutraceutical and in clinical trials. Recently, investigators of this study developed two OC taste-masked pharmaceutical formulations; a solid dispersion formulation with (+)-xylitol and an effervescent formulation, which maintained OC breast cancer (BC) suppressive effects [31,32]. The intake of xylitol large doses can cause gastrointestinal side effects due to its fermentation by gut microbiota [33,34]. Several powder pharmaceutical formulations reported with a large excess content of lecithin or lecithin-like substances claimed to provide bitter taste control [35]. A combination of soybean lecithin and magnesium aluminum silicate have been used to mask the unpleasant taste of talampicillin HCl [35]. The present study reports OC new powder formulations aimed to mask its irritative-pungent taste, avoid previous formulations weaknesses, and assess whether these formulations would still maintain the reported plain OC’s attenuation of AD progression. The OC powder formulation (OC-PF) can be easily filled into any of 0–4 size capsules feasible for oral administrations. The OC-solid dispersion formulation with erythritol (OC-SD) should not cause gastro-intestinal side effects of xylitol since erythritol will be readily systemically absorbed before reaching the intestinal tract. In addition, there is no reported OC pharmaceutical formulations validated to maintain its in vivo anti-AD activity. Thus, this study developed OC-PF and OC-SD oral formulations, with improved dissolution and maintained the in vivo activity on attenuation of Aβ pathology in 5xFAD transgenic mice. Both OC formulations suppressed C3AR1, a potential new molecular target and its downstream marker STAT3 in the brains of 5xFAD female transgenic mice, which may facilitate OC next-step development as a nutraceutical for amyloid pathology in AD.

## 2. Materials and Methods

### 2.1. Chemicals, Reagents, and Antibodies

Lactose, (±)-erythritol, aerosil-200, sodium lauryl sulfate, and magnesium stearate were purchased from Sigma Aldrich (St. Louis, MO, USA). Most of the Aβ and tau primary and secondary antibodies were purchased from Cell Signaling Technology (Beverly, MA, USA). p-Tau Ser199 was purchased from Abcam (Cambridge, MA, USA). C3AR1 which was acquired from Mybiosource, Inc. (San Diego, CA, USA).

### 2.2. Oleocanthal Isolation from Extra-Virgin Olive Oil 

Oleocanthal (OC) was extracted from EVOO (The Governor, batch #: 5-214000-242017) through a liquid–liquid extraction of EVOO with deionized water, and the aqueous layer was passed through Sorbtech (Sorbent Technology, Norcross, GA, USA) Sepabeads Resin Styrenic Adsorbent Sp-70-01 resin entrapment to remove water and residual fatty acid and entrap phenolics, which eluted with acetone later [36]. Final purification was achieved by liquid chromatography on SorbaDex Lipophilic Hydrophilic Gel Filtration Matrix LH-20 (Sorbent Technology, Norcross, GA, USA), isocratic elution with CH_2_Cl_2_ and the purity of OC (>99%) was confirmed through q^1^H NMR and HPLC analyses [36].

### 2.3. Preparation of OC Powder Mixture and Solid Dispersion Formulations

Preparation of OC powder formulation (OC-PF) started with adsorbing OC (10 mg) on the adsorbing agent Aerosil 200 (3 mg), which mixed with the lubricant magnesium stearate (0.5 mg) and the surfactant sodium lauryl sulfate (2 mg). Lactose (84.5 mg) then added as a binder and all ingredients uniformly blended. This mixture then passed over a 40-mesh screen and finally filled into a microcrystalline cellulose capsule shell. The hot melt fusion method was employed to prepare the solid dispersion of OC (OC-SD) with erythritol representing 1:7, *w*/*w*, OC: erythritol ratio [31]. Seven parts of erythritol were melted in a non-sticky pan by utilizing the IKA RCT basic hotplate at the temperature of 100 °C, and one part OC was gradually added [31]. The molten mixture was kept in the non-sticky pan and immediately cooled under dry N_2_ flow for 5 min. Upon samples solidification, they were grinded, stabilized, as well as stored at room temperature in a dark dry place [31].

### 2.4. Fourier Transform Infrared Spectroscopy 

A PerkinElmer Spectrum-Two™ FT-IR spectrometer (Waltham, MA, USA) was used to record the Fourier transform infrared (FT-IR) spectra of OC-PF and OC-SD formulations, plain OC, and placebo carriers. A diffuse reflectance cell was utilized to analyze each sample, without prior sample preparation, by directly compressing on an ATR crystal under appropriate compression conditions [31,32]. Each spectrum was scanned at a resolution of 4 cm^−1^ using the absorbance over the wavenumber range from 400–4000 cm^−1^. Each sample IR spectrum was acquired three times.

### 2.5. Powder X-ray Diffraction (PXRD) Analysis

PXRD patterns of OC-PF, OC-SD formulations versus their placebo formulations were analyzed through the application of a D8 DISCOVER X-ray diffractometer (Bruker Billerica, MA, USA). These analyses were carried out by Cu radiation (40 kV, 30 mA), at a scan range 20°–50°, and results were provided as intensity versus 2θ [31,32].

### 2.6. Scanning Electron Microscopy (ScEM)

OC-SD, OC-PF formulations, and their placebo formulations were placed on a brass stub using double-sided adhesive tape. They were then coated in a vacuum chamber with a thin layer of gold for 30s (Sputter coater, Edwards, S150A, England, UK) to execute electrically conductive fields. The images were then captured at an excitation voltage of 20 kV (Electron probe microanalyzer, JEOL, JXA-840A, Tokyo, Japan) to explore the surface morphology of each examined formulation.

### 2.7. In-Vitro Dissolution Study 

The dissolution profile of OC-PF, OC-SD, and plain non-formulated OC were carried out in a 100 mL simulated intestinal fluid (SIF, pH 6.8), without enzymes. This test was carried out through a USP type II dissolution apparatus (VK 7000, Varian Inc., Cary, NC, USA) at a paddle speed of 100 rpm [31]. The temperature of the dissolution medium was maintained at 37 ± 5 °C by the application of a Varian VK750 heater (Varian Inc., Cary, NC, USA). All formulations containing 10 mg OC and equivalent amount of pure OC were packed into a size 00 transparent hydroxypropyl methylcellulose (HPMC) capsule and inserted into the medium via an individual sinker. About 1 mL aliquot sample was withdrawn at time intervals of 10, 15, 30, 45, and 60 min, which was replaced with an equivalent amount of fresh dissolution medium. The collected samples were filtered, and OC content was analyzed by an HPLC system equipped with a UV/Visible variable wavelength detector at λ_max_ 230 nm (Shimadzu Scientific Instrument, Kyoto, Japan). Each experiment was repeated three times. A 20 µL sample of each time point was then injected into the Eclipse YD5 C18-RP analytical column (4.6 mm × 15 cm) at a flow rate of 1.0 mL/min using an isocratic acetonitrile–water (50:50) mixture as a mobile phase. Data acquisition and analysis were performed by Lab Solution™ chromatography software (Shimadzu Scientific Instrument, Kyoto, Japan).

### 2.8. Animals

All animal experiments were approved by the Institutional Animal Care and Use Committee (IACUC), University of Louisiana at Monroe, protocol number 18MAY-KES-04, approved on May 18, 2018, in accordance with the National Institutes of Health guidelines for animal care. 5xFAD mice exhibit human APP and PSEN1 transgenes with a total of five AD-linked mutations: the Swedish (K670N/M671L), Florida (I716V), and London (V717I) mutations in APP, and the M146L and L286V mutations in PSEN1 lined to familial AD. Four-week old 5xFAD female mice (16–18 g) were obtained from Jackson Laboratory-MMRRC (Stock number 008730, Bar Harbor, ME). Mice were acclimated at the animal housing facility of University of Louisiana-Monroe, College of Pharmacy and maintained under clean room conditions at 25 °C, 55–65% relative humidity, and a 12 h light/dark cycle for a week before experiments. Husk and excreta were taken away from the cages weekly. Mice had free access to purified drinking water and pelleted rodent chow (no. 7012, Envigo/Teklad, Madison, WI). Animals were orally dosed daily at 10 mg/kg OC in OC-PF, OC-SD, and vehicle control by 18G plastic (PTFE) oral feeding needles with stainless steel bite protector (VWR, Suwanee, GA, USA).

### 2.9. OC Formulations Orally Administered in 5xFAD Mice

After one-week acclimation, the 5xFAD female mice were randomly divided into three groups, *n* = 6 each. These groups were as follows: (i) vehicle-treated control group, (ii) OC-PF oral equivalent to OC 10 mg/kg treated group, (iii) OC-SD oral equivalent to OC 10 mg/kg treated group. All mice were orally dosed OC formulations using oral gavage. OC formulations were freshly dissolved in water, vortexed, and immediately used for dosing 6X/week at the same time of the day (noon) for 120 days. Animals were carefully observed individually every 4 h after the first dose for 24 h, and periodically over the 4-month treatment period. During this time, body weight and food consumption were monthly monitored. One week before sacrifice, the mice were trained daily for behavioral experiments. At the end of all experiments, mice were sacrificed and individual brain samples collected and stored at −80 °C for the further experiments.

### 2.10. Behavioral Studies

#### 2.10.1. Morris Water Maze

To conduct the Morris Water Maze test, a circular plastic tank (140 cm in diameter) was filled with water colored opaque with powdered non-fat milk and maintained at 22 °C ± 2 °C as previously described [37]. Mice were trained three times a day for three consecutive days so that at the end of the treatment all 5xFAD mice could find a way to a Plexiglas platform sub-merged in water from four different starting points within 60 s. If they were not able to find the platform within this period, they were manually guided to the platform and allowed to remain there for 15 s. Mice were trained to reach a training criterion of 20 s (escape latency). Animals’ movement were recorded and further analyzed using Any MazeTM Video Tracking System (Stoelting Co., Wood Dale, IL, USA). The parameters latency and swimming distance on the 4th day (experiment day after completion of 3-day training) were calculated and analyzed.

#### 2.10.2. Open-Field Activity

The Open-Field Activity test was performed to explore the locomotion and anxiety levels of the 5xFAD mice after completion of treatments [38]. During a 5-min test, mice were placed in an open field maze with field chamber (50 cm long × 50 cm wide), and activity was recorded from above and further analyzed using the Any MazeTM Video Tracking System (Stoelting Co., Wood Dale, IL, USA). Each 50 cm × 50 cm unit was digitally divided into 25 quadrants of equal size (9 central and 16 peripheral) through the application of the video tracking software. The nine central quadrants are collectively regarded as the center zone and the 16 peripheral quadrants as the peripheral zone. The total distance traveled, the average distance from the center, as well as the number of entries to, time spent in, and percentage distance traveled in the center, corner zones, and periphery were recorded and scored automatically.

#### 2.10.3. Elevated Plus Maze

The Elevated Plus Maze platform composed of two open and two closed arms (29 × 6 cm, each). Arms were elevated about 40 cm from the floor, which were used to evaluate the animal’s anxiety-related behavior in response to a potentially dangerous environment [38]. Each mouse was positioned in the center of the maze with their head facing towards the open arm and each experiment continued for 5 min. The time spent in each arm and the total distance traveled were recorded and further analyzed by Any MazeTM Video Tracking System (Stoelting Co., Wood Dale, IL, USA).

### 2.11. Western Blot Analysis

Collected whole brain tissues were homogenized in RIPA buffer (Qiagen Sciences Inc., Valencia, CA, USA) supplemented with protease and phosphatase inhibitors using an electric homogenizer and centrifuged at 15,000 g for 15 min to collect the supernatant. Protein concentrations were quantified by the BCA assay (Bio-Rad Laboratories, Hercules, CA, USA) and then protein samples were diluted with 5xLaemmli buffer. After boiling, 25 µg of protein was electrophoresed onto 10% SDS—polyacrylamide for protein separation, after that PVDF membranes were used to electro blot the gels. Blocking of membranes was done with 2% BSA in 10 mM Tris-HCl containing 50 mM NaCl and 0.1% Tween-20, pH 7.4 (TBST), and incubated with specific primary antibodies at dilution 1:1000 total tau (cell signaling, 46687T), phospho-tau Threonine 181 (cell signaling, 49561s), phospho-tau Serine 404 (cell signaling, 20194T), and phospho-tau Serine 199 (Abcam, ab1666747) overnight at 4 °C according to the manufacturer’s protocol. At the end of the incubation period, membranes were washed three times with TBST. Respective horseradish peroxide-conjugated secondary antibody in 2% BSA in TBST was used to incubate for 1 h at room temperature, and then rinsed with TBST five times. Visualization of blots was done through chemiluminescence according to the manufacturer’s instructions (Pierce, Rockford, IL, USA), along with the detection of proteins by the ChemiDoc XRS chemiluminescent gel imaging system. Analyses were executed through Image Lab software (Bio-Rad Laboratories). Visualization of β-tubulin was used to ensure equal sample loading in each lane. Western blotting experiments were carried out for each treatment group (*n* = 6 mice/treatment) and repeated three times [39].

### 2.12. Immunostaining

After sacrifice, brain tissues were immediately fixed in 10% neutral buffered formalin for 48 h. Then, those tissues were transferred to 70% ethanol, processed, and embedded in paraffin. Sectioning has been conducted at the AML Laboratories (Jacksonville, FL, USA). After de-paraffinization in xylene and graded ethanol, sections were boiled in citrate buffer (10 mM Sodium Citrate, pH 6) for 20 min, and then permeabilized in TBST solution for 15 min at room temperature. The sections were then stained with primary antibodies total Aβ (cell signaling, 8243T, 1:500), Aβ_42_ (cell signaling, 14974T, 1:500), Aβ_40_ (cell signaling, 12990S, 1:200), Aβ_43_ (cell signaling, 32098S, 1:50) diluted in blocking solution for 24 h at 4 °C. On the next day, sections were washed and stained with the secondary antibodies for 3 h prior to washing and mounting [14]. All images were captured in the Core Facility of LSUHSC-Shreveport, Louisiana at 10× magnification through Olympus iXplore CSU W1 Spinning Disk confocal microscope (Center Valley, PA, USA).

### 2.13. Congo-Red Staining

Congo red staining is considered as an accepted histochemical marker for the β-pleated-sheet structure of amyloid. Congophilic plaques were stained by subsequent application of Congo-red staining protocol implicated in detail in Wilcock et al. (2006) [40]. All animals brain tissue sectioning were deparaffinized by xylene and graded alcohols into tap water. Then, sections were immersed in alkaline sodium chloride. After 20 min, slides were immersed in alkaline Congo red solution for 20 min and differentiated through alcoholic potassium. In addition, sections were counterstained with alum hematoxylin and dehydrated through alcohols and xylene. All images were captured at 100× magnification using Olympus iXplore CSU W1 Spinning Disk confocal microscope (Center Valley, PA, USA).

### 2.14. Hematoxylin and Eosin Y (H&E) Staining

H&E staining was conducted at the AML Laboratories (Jacksonville, FL, USA), according to the manufacturer’s protocol. Briefly, paraffin-embedded brain tissues were deparaffinized through xylene, rinsed with graded alcohol, rehydrated by water, and finally, the tissue slides were stained with H&E and coverslipped with Permount [40]. All images were captured at 100× magnification through Olympus BX41 microscope (Center Valley, PA, USA).

### 2.15. Statistical Analysis

Values are expressed as means ± standard error of the mean (SEM) and analyzed using the statistical package for GraphPad Prism software version 8 through student t-test or differences among various treatment groups were determined by one-way analysis of variance (ANOVA) followed by post-hoc analysis by Tukey’s test; *p* < 0.05 was considered statistically significant.

## 3. Results

### 3.1. Characterization of OC-PF and OC-SD 

**Figure 1:**
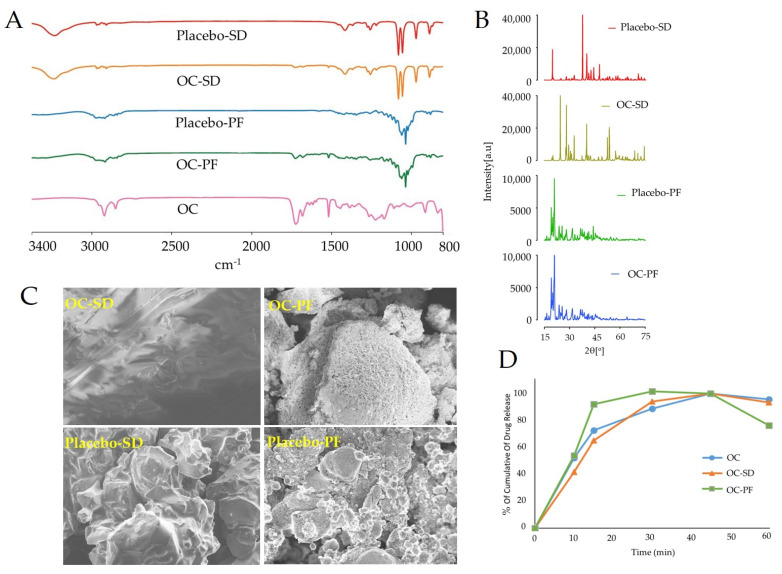
(**A**) FT-IR spectra of placebo-SD, OC-SD, placebo-PF, OC-PF, and OC. (**B**) Powder X-ray diffraction (PXRD) patterns demonstrating crystallite structural evolution of placebo-SD, OC-SD, placebo-PF, and OC-PF (from top to bottom). (**C**) Scanning electron micrographs of OC-SD, placebo-SD, OC-PF, and placebo-PF. (**D**) Comparison of the dissolution profile of plain OC with OC-SD and OC-PF in simulated intestinal fluid (pH 6.8) without enzymes.

The Fourier transform infrared spectroscopy (FTIR) data acquired for plain OC, OC-PF, placebo-PF, OC-SD, and placebo-SD and the results were analyzed (Figure 1A). The most characteristics IR fingerprint bands of OC are those at 1680 and 1723 cm^−1^ (C-1 and C-3 CHO aldehydes stretching, respectively) and were dominated in both OC-PF and OC-SD formulations. Other OC peaks were suppressed in OC-PF formulations because of the mixture of different excipients, and in the OC-SD formulation for complexation with erythritol. On the other hand, placebo and their corresponding formulations provided different spectra because of plausible entrapment of OC in the formulation carriers.

PXRD of OC-PF, placebo-PF, OC-SD, and placebo-SD were carried out to assess the changes of these formulation crystalline forms through the alteration of their diffraction pattern and phase purity (Figure 1B). All formulations exhibited subsequent sharp reflections indicating the crystallinity behavior, while the oily properties of OC did not change the original characters of any crystalline ingredient through the formulation process. However, different diffraction patterns for OC-SD and placebo-SD were observable. The PXRD pattern of OC-SD demonstrated the crystalline nature with several prominent high intensity peaks observed at approximately 2 h ¼ 24.6, 28.5, 32.5, 40, 52.6, 53.8 and other less prominent peaks at 2 h ¼ 14.5, 29.6, 37.3, 57.5, 68.8, 71.5, 74.5, and 75.5. In addition, prominent peaks of high intensity at approximately 2h ¼ 37.5 and other less prominent peaks at 2 h ¼ 19.7, 40, 41.3, 42.5, 44.2, 47.7, and 70.5 were observed in placebo-SD. These finding suggested that OC inclusion generated crystallinity transformation through the SD with erythritol and provided different crystal lattice morphology. On the other hand, the PXRD pattern of OC-PF exhibited a crystalline nature with a prominent peak of high intensity at approximately 2h ¼ 20 and 19, and four other less prominent peaks at 2 h ¼ 24, 25.5, 28, and 31.5. Meanwhile, placebo-PF indicated crystalline nature with prominent peaks of high intensity at approximately 2 h ¼ 20.5 and 19, and five other less prominent peaks at 2 h ¼ 23.7, 25.5, 28, 31.5, and 44.5. Observed elimination and/or significant reduction in peak intensity was detected in OC-PF compared to placebo-PF (Figure 1B). This attenuation in mixture crystallinity was assumed as a drug solubility and dissolution-enhancing factor [41,42,43].

The micro and macrostructural surface morphology of the OC-PF, placebo-PF, OC-SD, and placebo-SD were investigated by scanning electron microscopy (ScEM) (Figure 1C). OC-PF and OC-SD formulations displayed a noticeable alteration of the surface structure compared to their corresponding placebo. In both formulations, the formulation carriers showed significant improvement in surface morphology, appearing spherical and smooth surface with smoothly curved sides, and a more cohesive arrangement. In contrast, the placebo demonstrated sub-angular–sub-rounded particles with a smooth surface of fine particles and wide gaps.

### 3.2. In Vitro Dissolution Study

The dissolution profiles of plain OC, OC-PF, and OC-SD in simulated intestinal fluid (SIF, pH 6.8) were compared (Figure 1D). Both OC-PF and OC-SD provided significant OC dissolution enhancement compared to the non-formulated plain OC. Within 10 min of the dissolution study, approximately 50% of OC in OC-PF and OC-SD was released and dissolved, whereas only 40% of plain OC was dissolved (Figure 1D). At 30 min, OC-PF was completely dissolved, whereas OC-SD was dissolved 85% and OC showed 80% dissolution at this time point.

### 3.3. OC Formulation Treatments Effects on Mice Body Weight and Food Intake

Our study demonstrated that over a 4-month oral administration of OC-SD or OC-PF, there was no significant change in 5xFAD mice body weight compared to vehicle control group (Supplementary Figure S1A). Weekly monitoring of average food consumed by animals over the 4-month treatment period indicates reduced food intake by animals treated with the OC-PF group (253 g) and OC-SD (228 g) as compared to the vehicle control-treated group (314 g) (Supplementary Figure S1B).

### 3.4. OC Formulation Treatments Improved 5xFAD Mice Spatial Learning and Memory Ability

**Figure 2:**
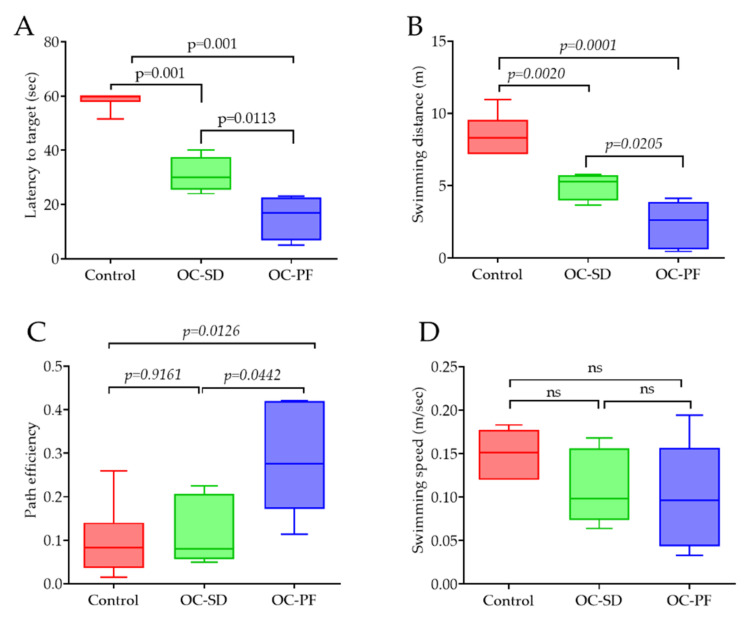
Spatial learning and memory ability effects of OC-PF and OC-SD on 5xFAD mice using the Morris Water Maze (MWM) model. (**A**) Latency to target, F (2, 15) = 84.93, *p* < 0.05. (**B**) Swimming distance, F (2, 15) = 30.51, *p* < 0.05. (**C**) Path efficiency, F (2, 15) = 6.072, *p* < 0.05. (**D**) Swimming speed, F (2, 15) = 1.924, “ns”: non-significant, for 5xFAD mice in MWM task. Data analyzed using one way ANOVA followed by post-hoc Tukey test and presented as mean ± SEM (*n* = 5 per group).

The effects of 4-month oral use of OC-PF, OC-SD, and vehicle on 5xFAD female mice spatial learning and memory ability were assessed. The Morris Water Maze (MWM) experiment was used. MWM included the latency (the time a mouse takes to find the platform(s), swimming distance (cm), swimming speed (cm/s), and path efficiency, which were analyzed after three consecutive training days. In latency to the target, both OC-PF and OC-SD-treated mice (15.48 ± 4.19 and 31 ± 3.31, respectively) showed significant shorter latency time compared to the vehicle control-treated mice (58.60 ± 1.4 s) (Figure 2A). OC-PF-treated mice also had significantly lower latency time to target (*p* = 0.0113) compared to OC-SD. In swimming distance, both OC-PF and OC-SD-treated groups significantly attenuated mice swimming distance, compared to the vehicle control group (Figure 2B). Moreover, long-term treatments with OC-SD and OC-PF improved the spatial learning in the MWM test, compared to the vehicle control as indicated by the significantly shorter goal latency times or path efficiency (Figure 2C). In addition, the MWM experiment also revealed no significant change in swimming speed among the vehicle control and treatment groups, which excludes the incidence of motor changes (Figure 2D).

**Figure 3:**
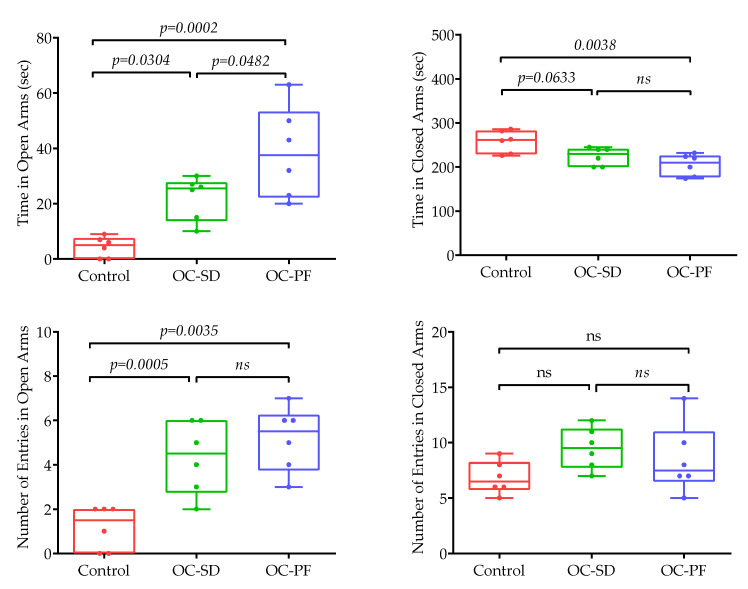
The effects of OC-PF and OC-SD treatments in 5xFAD mice using the elevated plus maze test. (**A**) Mice time in open arm (F (2, 15) = 14.99, *p* < 0.05). (**B**) The number of tested mice entries in open arms F (2, 15) = 13.82, *p* < 0.05). (**C**) The time taken by tested mice in closed arm (F (2, 15) = 7.80, ns and *p* < 0.05). (**D**) The number of tested mice entries in closed arm F (2, 15) = 2.099, ns. Resuts analyzed using one-way ANOVA followed by post-hoc Tukey test and presented as mean ± SEM (*n* = 6 per group). “ns”: non-significant.

The anxiety-related behavior of 5xFAD mice were investigated after completion of treatments with either OC-PF, OC-SD, or vehicle control. Two different behavioral tests used; (i) The elevated plus maze (EPM) task (Figure 3) and (ii) The open field (OF) arena (Supplementary Figure S2). In Figure 3A, the open arms, neither the amount of time spent nor the number of entries in open arms significantly increased in OC-PF and OC-SD-treated group mice versus the vehicle control group. This pattern suggested that both treatment groups have reduced levels of anxiety compared to the vehicle control. 

In the open field experiment, the measure of baseline locomotion in a novel environment, several parameters considered including the number of entries in corner/center/peripheral zones, time spent in these area, distance, and average speed. Supplementary Figure S2 suggests the lack of significant differences in OC-PF and OC-SD-treated animals compared to the vehicle control group after 5 min of open field exploration. There were no significant differences observed between the vehicle control and both treatment groups mice for the number of entries and amount of time spent at the different zone. There was no noticeable effect in the total distance traveled and average speed in each zone by both treatment groups versus the vehicle control mice. 

### 3.5. OC Formulations Attenuated Aβ Plaque Accumulation in 5xFAD Mice

**Figure 4:**
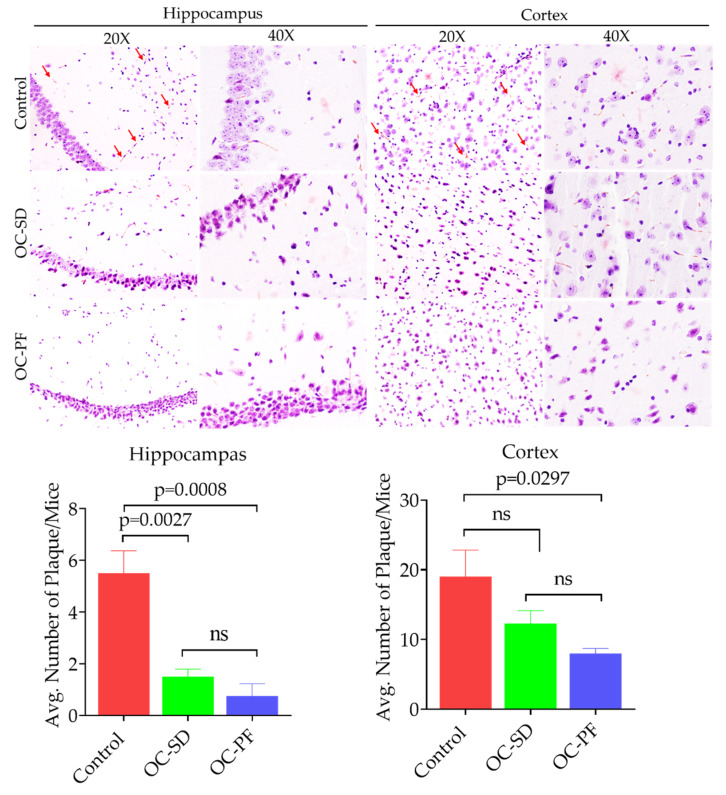
(**A**) OC-SD and OC-PF treatments attenuated the Aβ plaque deposition evidenced by Congo red staining of 5xFAD mouse brain sections (*n* = 4 mice/group). Representative brain sections for Aβ plaque deposition in hippocampus and cortex. (**B**) Quantitative analysis of average Aβ plaque count in tested mice brain hippocampus sections, F (2, 9) = 18.41, *p* < 0.05 and (**C**) in tested mice brain cortex sections, F (2, 9) = 4.973, ns and *p* < 0.05. Data were analyzed using one-way ANOVA followed by post-hoc Tukey test and presented as a mean ± SEM (*n* = 4 per group). “ns”: non-significant.

**Figure 5:**
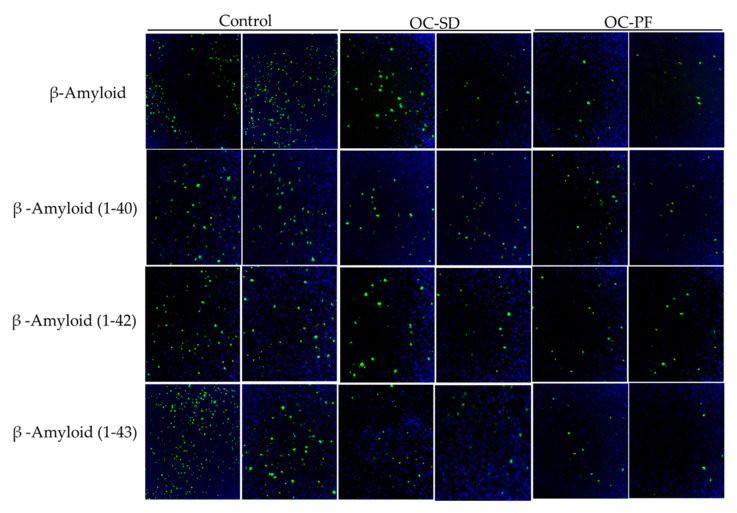
Representative 5xFAD mouse brain cortex sections (*n* = 4 mice/group) stained with Aβ antibody (green) for total Aβ deposition, AB_40_, AB_42_, AB_43_; DAPI (blue) was used to stain the nuclei.

The effect of oral administration of OC-PF, OC-SD, and vehicle control on Aβ production in the 5xFAD mice brain were evaluated by using Congo Red staining and Aβ marker through immunofluorescence staining (Figure 4,Figure 5). In Congo Red staining assay, there was high intensity of total Aβ deposits in the cortex, compared to the hippocampus of 5xFAD mouse brains. One-way ANOVA analysis further confirmed that OC-PF treatment significantly reduced the intensity of Aβ deposition compared to the vehicle control, both in hippocampus (*p* = 0.008) and cortex (*p* = 0.0297) regions (Figure 4A–C). OC-SD significantly reduced the Aβ depositions (*p* = 0.0027) at the hippocampal region versus the vehicle control group. OC-SD treatment attenuated the overall Aβ depositions but not to the statistically significance level in the cortex region (Figure 4A–C). However, both the OC-PF and OC-SD treatment showed modulatory effects on the Aβ accumulation in both hippocampus and cortex regions of 5xFAD mouse brains versus the vehicle control group. 

**Figure 6:**
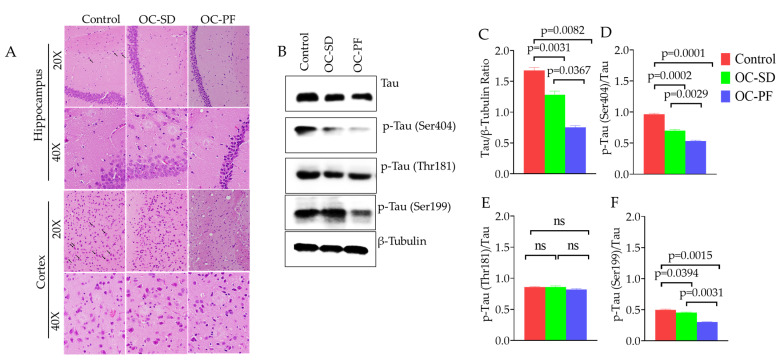
Effects of OC-PF and OC-SD treatments on tau phosphorylation in 5xFAD mouse brains. (**A**) Representative 5xFAD mouse brain cortex and hippocampus sections stained with H&E. (**B**) OC-PF and OC-SD treatments effect on the levels of total and tau phosphorylation at amino acid residues Ser404, Thr181, and Ser199 in 5xFAD female mice brain homogenates. (**C**–**F**) Scanning densitometric analysis of blots for total tau (F (2, 6) = 89.74, *p* < 0.05), p-Tau (Ser404) (F (2, 6) = 15.3, *p* < 0.05), p-Tau (Thr181) (ns), and p-Tau (Ser199) (F (2, 6) = 36.90, *p* < 0.05), respectively. Scanning densitometry was obtained for each blot, carried out in triplicate and the integrated optical density of each band was normalized with the corresponding density found for β-tubulin in the same blot. Data are presented as mean ± SEM. Statistical analysis was determined by one-way analysis of variance (ANOVA) followed by post-hoc analysis by Tukey’s test; *p* < 0.05 was considered statistically significant, *n* = 6 mice/group. “ns”: non-significant.

The effects of OC-PF and OC-SD treatments against Aβ plaque deposition in 5xFAD mouse brains at the cortex region further investigated by immunostaining of different Aβ antibodies. We observed that OC-PF and OC-SD reduced the accumulation of the total Amyloid β (Figure 5). Interestingly, immunofluorescence assay indicated that OC-PF and OC-SD notably reduced the levels of Aβ-40, Aβ-42, and Aβ-43, compared to the vehicle control group, which was consistent with the Congo Red staining findings (Figure 5). Consistent with these finds, the H&E staining of both formulations exhibited notable Aβ plaque reduction compared to control (Figure 6A).

### 3.6. OC Formulations Effect on Tau Pathology in 5xFAD Mice

The effects of OC-PF and OC-SD treatments on tau phosphorylation activity were also investigated by Western blotting of collected 5xFAD mouse brains (Table 1). Significant downregulations of total tau levels observed in OC-SD (*p* = 0.03) and OC-PF (*p* = 0.008)-treated mice compared to the vehicle control 5xFAD mice brain homogenate samples (Table 1 and Figure 6B, C). The phosphorylated-tau Ser404 and Ser199 levels in 5xFAD mouse brain homogenates significantly suppressed by OC-PF (*p* = 0.004 and *p* = 0.001, respectively), whereas OC-SD treatments suppressed p-Tau Ser404 (*p* = 0.019) compared to the vehicle control group (Figure 6D, F). In addition, OC-PF and OC-SD showed modest effect on the p-Tau Thr181 (Table 1 and Figure 6E).

### 3.7. Effects of OC Formulation Treatments on C3AR1 and STAT3

**Figure 7:**
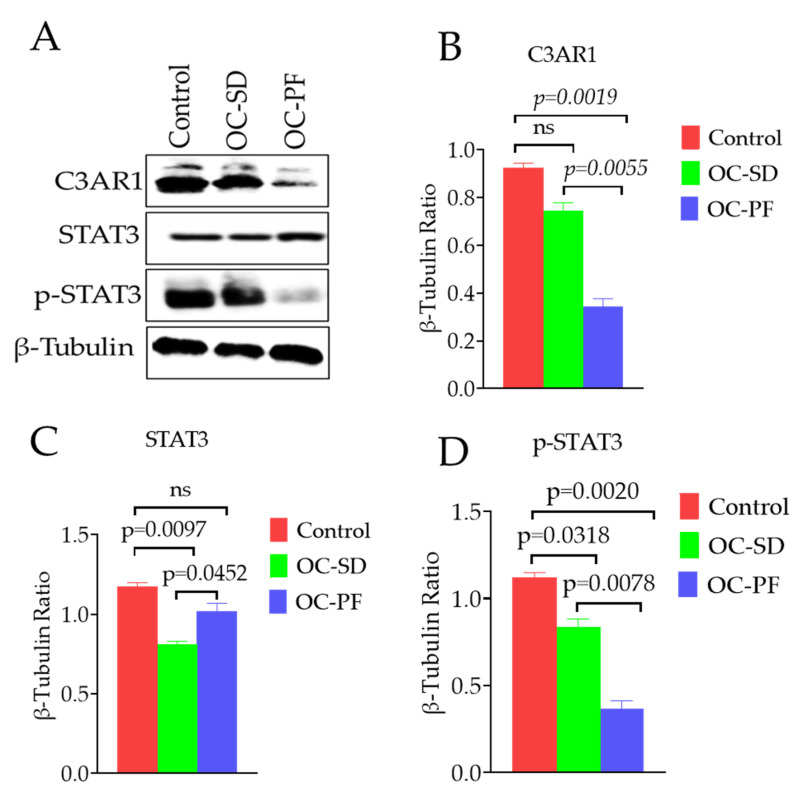
OC-PF and OC-SD treatment effects on the expression level of C3AR1, total and activated downstream target STAT3 in 5xFAD mouse brains. (**A**) Western blot showing the OC-PF and OC-SD treatments effect on the expression level of C3AR1, STAT3, and p-STAT3 in 5xFAD brain homogenates. (**B**–**D**) Scanning densitometric analysis of the blot for C3AR1 (F (2, 6) = 93.70, *p* < 0.05, STAT3 (F (2, 6) = 29.05, *p* < 0.05), and p-STAT3 (F (2, 6) = 88.00, *p* < 0.05), respectively. Scanning densitometry was executed for each blot, conducted in triplicate and the integrated optical density of each band was normalized with the corresponding density found for β-tubulin in the same blot. Data are presented as mean ± SEM. Statistical analysis was determined by one-way analysis of variance (ANOVA) followed by post-hoc analysis by Tukey’s test; *p* < 0.05 was considered statistically significant, *n* = 6 mice/group. “ns”: non-significant.

The effects of OC-PF and OC-SD treatments was assessed on the complement C3-C3aR signaling in 5xFAD brain homogenates using Western blots analysis of the C3AR1 antibody. OC-PF treatment significantly decreased the expression level of C3AR1 compared to the vehicle control. In contrary, the OC-SD treatment showed only a slight overall C3AR1 expression level reduction and was statistically not significant (Figure 7A,B). The STAT3 signaling pathway was also investigated since the p-STAT3 assumed as the downstream effector of C3AR1. While the OC-PF did not affect the total STAT3, the OC-SD reduced the total level of STAT3 compared to the vehicle control group (Figure 7C). Interestingly, both treatment groups OC-PF and OC-SD significantly downregulated the activation of p-STAT3, compared to the vehicle control group mice (Figure 7D).

## 4. Discussion

About 121 therapeutic agents are currently in clinical trials for prospective treatment of AD [44]. Most of these drug candidates are disease-modifying agents targeting pathways other than amyloid or tau specified [44]. Although researchers are trying to focus the mainstream AD drug discovery and development efforts on the components or therapies directly involved in AD pathology, compelling evidence points to the low success rate of AD therapeutics because of toxicity and weak clinical efficacy concerns [45,46]. Thus, the current drugs used to control AD are unsatisfactory, reflecting a dire need to target the mainstream of AD and develop more effective and safer drugs with minimal toxicity. On the other hand, interestingly, OC has been already exhibited in vitro and in vivo modulating β-amyloid, tau phosphorylation, and neuroinflammation activities in different models. A recent study showed the high safety profile of OC acute single oral dose in a Swiss albino mice model [47]. Unfortunately, one of the major obstacles for the application of OC as a prospective nutraceutical are its irritant, pharyngeal pungent, and astringent taste, similar to the NSAID drug-ibuprofen. These effects are attributed to OC activation of the transient receptor potential cation channel subtype A1 (TRPA1) receptor [19,48,49,50]. Other challenges associated with OC therapeutic applicability are its potential chemical instability imparted by its reactive C-3 aldehyde, hydrolysable linear ester group, and poor water solubility. In the present study, developed OC formulations prospectively overcame its oral delivery problem and maintained its positive amyloid pathogenesis activities in a mouse model. The OC-PF made by mixing OC with FDA-approved inactive pharmaceutical excipients while the OC-SD formulated by the solid-dispersion of the oily OC with heat-melted sugar alcohol erythritol crystals. Erythritol was preferred over the previously reported xylitol because it is expected to produce less gastrointestinal side effects [31,33,34,35]. Both formulations are physically and chemically characterized by FTIR, ScEM, PXRD, and dissolution studies. An improved formulation dissolution profile was clearly exhibited by both formulations over the plain OC.

The activity of OC-PF and OC-SD on AD-associated amyloid pathogenesis progression was assessed in 5xFAD female mice model after 4-month oral 10 mg/kg, 6X/week treatments. In behavioral tests, both formulations showed learning improvement in the Water Maze test. OC-PF significantly decreased the latency to target in the MWM test and the time spent in the open arms in the EPM test compared to OC-SD. Both formulations significantly mitigated the Aβ plaques aggregation in the 5xFAD female mouse brains and this result was consistent with the immunofluorescence study results of the total Aβ, Aβ40, Aβ42, and Aβ43 (Figure 5, Supplementary Figure S3). Further, H&E staining of both formulations showed attenuation of Aβ plaque (Figure 6A). Overall, both OC-PF and OC-SD treatments ameliorated the pathological features and behavioral deficits in 5xFAD mice by attenuating the level of Aβ deposition.

Among the 36 to 43 amino acids of Aβ, the effects of soluble Aβ40 and insoluble Aβ42 on AD were the most extensively investigated. Aβ42 is regarded as the major constituent of the plaques produced via the cleavage of Aβ precursor protein (APP) by β- and γ-secretases. Aβ42 assumed more toxic than Aβ40 due to its higher probability to aggregate [51]. Literature validated the elevated levels of Aβ40 and Aβ42 in different mouse models, which lead to neuronal deficits and long-term potentiation impairment [52,53,54]. 

Recently, numerous studies proved Aβ43 as a biomarker in early onset of AD. Aβ43 differs from Aβ42 by a single C-terminal threonine residue [55]. Like other Aβ, Aβ43 generated from an alternative γ-secretase cleavage pathway of the APP. An increasing body of evidence indicates that the neurotoxicological effect of Aβ43 is as high as Aβ42 or Aβ40 in different models of AD, including mouse models and humans [56]. Actually, some mouse models like the ones with PSEN1 mutations associated with familial AD or mutant APP transgenic mice exhibited overproduction of Aβ43 more than Aβ42. On the other hand, cerebral deposition of Aβ43 is frequently observed in both sporadic and familial AD [57,58]. In the current study findings, overall significant reduction of total Aβ, Aβ40, Aβ42, and Aβ43 aggregation noted in 5xFAD mouse brains via the use of both OC-PF and OC-SD formulations, highlighting their translational potential for therapeutic application against the AD-associated amyloid pathogenicity. Both formulations significantly decreased the p-Tau (Ser199) levels as compared to the control, but the levels of this specific p-Tau protein was significantly lowered in OC-PF treated mice versus those treated with OC-SD (Table 1) [59]. Similarly, the total tau and p-Tau (Ser404) downregulation in OC-PF-treated mice was nearly 2-fold those treated with OC-SD (Table 1) [59]. This clearly suggests the superior activity of OC-PF over OC-SD.

Recently, C3AR1, with proven dysregulation in AD, was hypothesized as a potential molecular target to modulate neuroinflammation without adversely affecting other critical complement processes [13,14]. The role of the complement C3AR in the immune network and neuronal tau pathology was also validated [14]. The effects of OC-PF and OC-SD on C3AR1 expression in 5xFAD mice brain samples were explored by Western blotting. Mice treated with OC-PF showed significant inhibition of C3AR1 expression. The OC-SD also reduced the expression of C3AR1 in the 5xFAD mouse brains but this effect was not statically significant. Several studies revealed the incidence of activation and localization of proteins of the complement cascade, especially C3a in and around amyloid plaques tau pathology [59,60]. Considering the validated anti-AD activity of OC, a key finding of this study was the C3AR1 expression attenuation in the 5xFAD mouse brains by OC-PF and OC-SD compared to the vehicle control. In 2018, Litvinchuk et al. showed the higher expression of C3 and C3AR1 in human brains with hyperphosphorylated tau and identified p-STAT3 as the direct downstream effector of the C3-C3AR signaling with detrimental effects in AD [14]. Thus, exploring the effect of OC-PF and OC-SD on the total and activated STAT3 levels in treated 5xFAD mouse brains was justified. Effects of OC-PF and OC-SD treatments were obvious through the downregulation of the p-STAT3 level compared to vehicle control-treated mouse brains (Figure 7). Overall, OC-PF showed better activity than OC-SD, which may justify its superior potential for future use to control AD-associated amyloid pathology as a delivery system for OC. Progressive evidence supports the pivotal role of STAT3 as an upstream regulator of the pathogenesis of AD-related memory impairment [14,61,62,63]. STAT3 phosphorylation is also augmented by the complement C3 [14]. On the other hand, STAT3 phosphorylation inhibition reduced the learning and memory impairments in 5xFAD mice [62,63,64].

## 5. Conclusions

This study introduced and characterized two novel oral formulations of OC with improved dissolution and pharmacodynamics pattern. These formulations maintained amyloid pathogenesis suppressive activity displayed by reducing the β-amyloid accumulation in 5xFAD mouse brains. Additionally, strong evidence implicates a pivotal role of the complement C3AR1-STAT3 signaling axis in mediating the molecular mechanism of the OC formulation treatments used for dosing 5xFAD female mice. Results posit the OC-PF as a prospective nutraceutical/dietary supplement appropriate for future use by patients, survivors, and elderly people at high risk of AD to control amyloid pathogenesis.

## Figures and Tables

**Table 1:** Western blotting-based quantitative downregulation of total and selected phosphorylated tau levels in 5xFAD female mouse brains in response to 4-month oral treatments by OC formulations.

	% Tau Downregulation	
	OC-SD	OC-PF
Total Tau	23.4	55.1
p-Tau(Ser404)	27.8	44.9
p-Tau(Thr181)	0.1	4.7
p-Tau(Ser199)	8.8	39.4

## Supplementary Materials

The following are available online at https://www.mdpi.com/article/10.3390/nu13051702/s1, Figure S1: Overview monitoring of the effects of OC-PF and OC-SD four-month treatments course on body weight and food consumption in 5xFAD female mice. Figure S2: Comparison of the effects of four-month OC-PF and OC-SD treatments course in 5xFAD mice versus vehicle control using the open-field test for general exploration and anxiety assessments. Figure S3. Representative 4X and 40X magnification of 5xFAD mouse brain cortex sections (*n* = 4 mice/group) stained with Aβ antibody (green) for total Aβ deposition and AB42, DAPI (blue) was used to stain the nuclei.

## Abbreviations

AD	Alzheimer’s Disease
Aβ	Aβ-amyloid
ADDLs	Aβ-Derived Diffusible Ligands
C3AR1	Complement component 3a receptor 1
EVOO	Extra-virgin olive oil
IHC	Immunohistochemistry
MWM	Morris Water Maze
NFT	Neurofibrillary tangles
NS	Non-significant
OC-PF	Oleocanthal Powder Formulation
OC-SD	Oleocanthal Solid Dispersion
PXRD	Powder X-ray Diffraction
ScEM	Scanning electron microscopy
SEM	Standard error of the mean
STAT3 TRPA1	Signal transducer and activator of transcription 3 Transient receptor potential cation channel subtype A1 receptor
