# Supplementary material for: (-)-Oleocanthal Nutraceuticals for Alzheimer’s Disease Amyloid Pathology: Novel Oral Formulations, Therapeutic, and Molecular Insights in 5xFAD Transgenic Mice Model
Source: Nutrients. 2021 May 18;13(5):1702. doi: 10.3390/nu13051702 (PMC8157389; doi:10.3390/nu13051702)
Supplement: Supplementary file 1 [file nutrients-13-01702-s001.zip › nutrients-1166652-supplementary.pdf]

nutrients

MDPI

Research Article

Supporting Information

(-)-Oleocanthal Nutraceuticals for Alzheimer's Disease Amyloid Pathology: Novel Oral Formulations, Therapeutic, and Molecular Insights in 5xFAD Transgenic Mice Model

Afsana Tajmim,[1] Areli K. Cuevas-Ocampo,[2] Abu Bakar Siddique,[1] Mohammad Qusa,[1] Judy Ann King,[2] Khaldoun S. Abdewahed,[1] Jafrin Jobayer Sonju,[1] and Khalid A. El Sayed[1,*]

[1] School of Basic Pharmaceutical and Toxicological Sciences, College of Pharmacy, University of Louisiana at Monroe, 1800 Bienville Drive, Monroe, LA 71201, USA; tajmima@warhawks.ulm.edu (A.T.); siddique@ulm.edu (A.B.S.); qusamh@warhawks.ulm.edu (M.Q.); abdelwks@warhawks.ulm.edu (K.A.W.); sonjujj@warhawks.ulm.edu (J.J.S.); elsayed@ulm.edu (K.A.E.S.)

[2] Department of Pathology and Translational Pathobiology, LSU Health Shreveport, 1501 Kings Highway, Shreveport, LA, 71103, USA; acueva@lsuhsc.edu (A.K.C.O.); jkin12@lsuhsc.edu (J.K.)

* Correspondence: elsayed@ulm.edu; Tel.: +1-318-342-1725

Citation: Lastname, F.; Lastname, F.; Last-name, F. Title. *Nutrients* **2021**, *13*, 1702. https://doi.org/10.3390/nu13051702

Received: date
Accepted: date
Published: 18 May 2021

Publisher's Note: MDPI stays neutral with regard to jurisdictional claims in published maps and institutional affiliations.

cc BY

Copyright: © 2021 by the author. Licensee MDPI, Basel, Switzerland. This article is an open access article distributed under the terms and conditions of the Creative Commons Attribution (CC BY) license (http://creativecommons.org/licenses/by/4.0/).

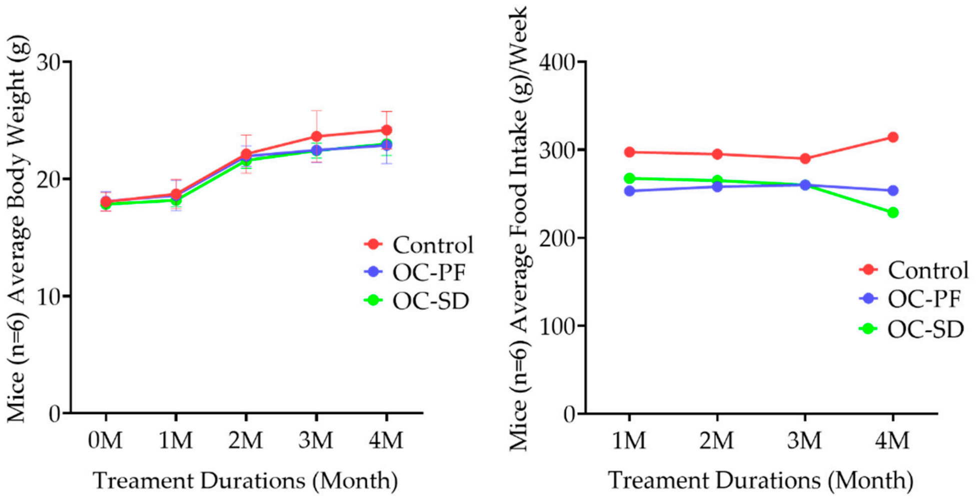

Supplementary Figure S1. The effects of OC-PF and OC-SD four-month treatments course in 5xFAD female mice body weight and food consumptions. (A) Average body weight of the control, OC-SD, and OC-PF-treated 5xFAD mice. **(B)** Average food intake per week of the control, OC-SD and OC-PF-treated 5xFAD mice.

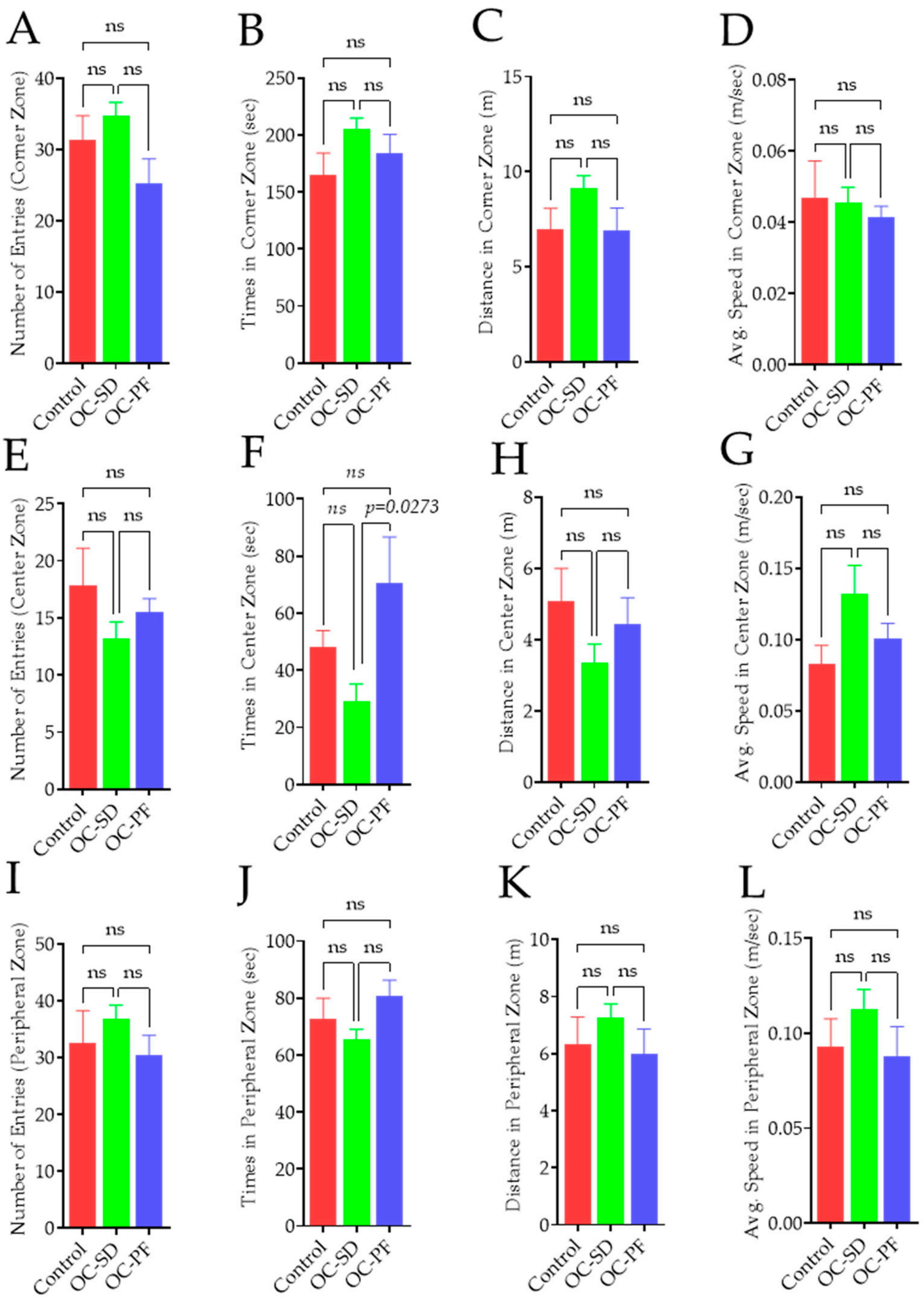

Supplementary Figure S2. The effects of OC-PF and OC-SD four-month treatments course in 5xFAD female mice using the open field test for general exploration and anxiety. (A-D) Number of entries to, time spent, distance travelled and average speed in the corner zone. **(E-H)** Number of entries to, time spent, distance travelled and average speed in the center zones. **(I-L)** Number of entries to, time spent, distance travelled and average speed in the peripheral zone. There was no effect of OC-SD and OC-PF in compared to control on the number of entries to, time spent and percentage distance travelled in the center zone. OC-PF spent more time in the center zone than OC-SD (p =0.0273), but there was no effect of OC-SD and OC-PF in compared to control on time spent, number of entries and distance travelled in the

corner zones. There was no effect of OC-SD and OC-PF in compared to control on the number of entries to, time spent and distance travelled in the periphery zone. Individual datapoints are presented on the plot. Data are presented as mean ±the standard error of mean(SEM). Data analyzed using One-way ANOVA followed by post-hoc Tukey test and presented as mean ± SEM (n = 6 per group).

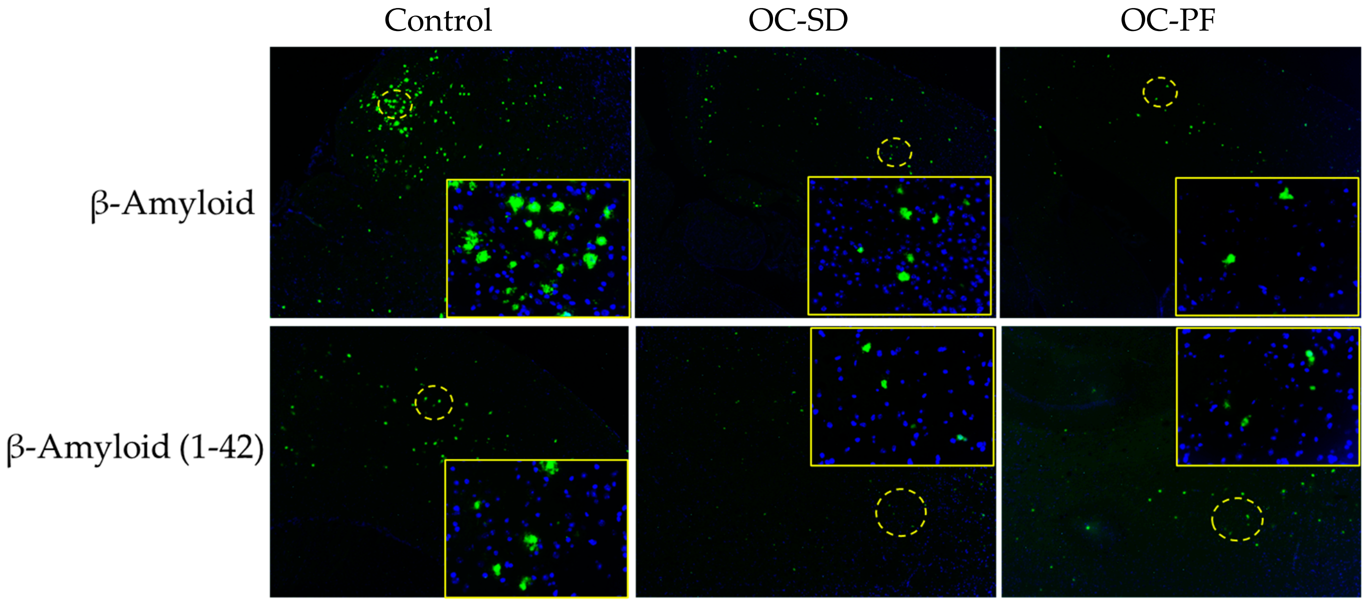

Supplementary Figure S3. Representative IHC photographs for 5xFAD female mice brain cortex sections (n = 4 mice/group) stained with Aβ antibody (green) for the total Aβ deposition and AB42. DAPI (blue) was used to stain the nuclei. Images are available at 4X and 40X (square) magnification for selected (circled) areas.
